# Supplementary material for: A potential marker of radiation based on 16S rDNA in the rat model: Intestinal flora
Source: PLoS One. 2023 Aug 1;18(8):e0286026. doi: 10.1371/journal.pone.0286026 (PMC10393172; doi:10.1371/journal.pone.0286026)
Supplement: Attachment — Submitted filename: PONE-D-22-28249_Comments.pdf [file pone.0286026.s002.pdf]

PLOS ONE

A potential marker of radiation based on 16S rDNA in the rat model: intestinal flora

--Manuscript Draft--

**Manuscript Number:**	PONE-D-22-28249
**Article Type:**	Research Article
**Full Title:**	A potential marker of radiation based on 16S rDNA in the rat model: intestinal flora
**Short Title:**	A potential marker of radiation based on 16S rDNA in the rat model
**Corresponding Author:**	YongQi Liu<br>Gansu University of Traditional Chinese Medicine<br>Lanzhou, 甘肃 CHINA
**Keywords:**	X-ray; radiation; 16S rDNA; intestinal flora dysbiosis
**Abstract:**	The gastrointestinal microbiota plays an important role in the function of the host intestine. However, little is currently known about the effects of irradiation on the microorganisms colonizing the mucosal surfaces of the gastrointestinal tract. The aim of this study was to investigate the effects of X-ray irradiation on the compositions of the large intestinal microbiotas of the rat. The gut microbiotas in control mice and mice receiving irradiation with different dose treatment were characterized by high-throughput sequencing of the bacterial 16S rDNA gene and their metabolites were detected by gas chromatography-mass spectrometry. Unexpectedly, the diversity was increased mildly at 2Gy irradiation, and dose dependent decreased at 4Gy, 6Gy, 8Gy irradiation. The phyla with large changes in phylum level are Firmicutes, Bacteroides and Proteobacteria; the abundance ratio of Firmicutes/Bacteroides is inverted; and when 8Gy is irradiated, the phylum abundance level was significantly increased. At the genus level, the abundance levels of Phascolarctobacterium, Ruminococcaceae and Lachnospiraceae increased at 2Gy irradiation, and significantly decreased at 4Gy, 6Gy, and 8Gy irradiation; the abundance level of Prevotellaceae diminished at 2Gy irradiation, and enhanced at 4Gy, 6Gy, 8Gy irradiation; The abundance level of Violet bacteria (Christenellaceae) and Lactobacillus attenuated in a dose-dependent manner; Lachnoclostridium enhanced in a dose-dependent manner; Bacteroides was in 4Gy, 6Gy, 8Gy The abundance level increased significantly during irradiation; the abundance level of Shigella (Escherichia-Shigella) only increased significantly during 8Gy irradiation. Lefse predicts that the biomarker at 0Gy group is Veillonellaceae, the biomarker at 2Gy group is Firmicutes, the biomarkers at 4Gy group are Dehalobacterium and Dehalobacteriaceae, the biomarkers at 6Gy group are Odoribacter, and the biomarkers at 8Gy group are Anaerotruncus , Holdemania, Proteus, Bilophila, Desufovibrionales and Deltaproteobacteria. Overall, the data presented here reveal that X-ray irradiation can cause imbalance of the intestinal flora in rats; different doses of irradiation can cause different types of bacteria change . Representative bacteria can be selected as biomarkers for radiation damage and repair.
**Order of Authors:**	YongQi Liu
	Li-Ying Zhang
	Yang-Yang Li
	xiaomin Xu
	zhiming miao
	Ting Zhou
	Yi-Ming Zhang
**Additional Information:**	
**Question**	**Response**
**Financial Disclosure**	NO - Include this sentence at the end of your statement: The funders had no role in study design, data collection and analysis, decision to publish, or preparation of the

Powered by Editorial Manager® and ProduXion Manager® from Aries Systems Corporation

Enter a financial disclosure statement that describes the sources of funding for the work included in this submission. Review the submission guidelines for detailed requirements. View published research articles from *PLOS ONE* for specific examples.<br><br>This statement is required for submission and **will appear in the published article** if the submission is accepted. Please make sure it is accurate.<br><br>**Unfunded studies**<br>Enter: *The author(s) received no specific funding for this work.*<br><br>**Funded studies**<br>Enter a statement with the following details:<br>• Initials of the authors who received each award<br>• Grant numbers awarded to each author<br>• The full name of each funder<br>• URL of each funder website<br>• Did the sponsors or funders play any role in the study design, data collection and analysis, decision to publish, or preparation of the manuscript?<br>• **NO** - Include this sentence at the end of your statement: *The funders had no role in study design, data collection and analysis, decision to publish, or preparation of the manuscript.*<br>• **YES** - Specify the role(s) played.<br><br>* typeset	manuscript.
**Competing Interests**<br><br>Use the instructions below to enter a competing interest statement for this submission. On behalf of all authors, disclose any competing interests that could be perceived to bias this work—acknowledging all financial support and any other relevant financial or non-financial competing interests.<br><br>This statement is required for submission and **will appear in the published article** if the submission is accepted. Please make	NO authors have competing interests<br>Enter: The authors have declared that no competing interests exist.

sure it is accurate and that any funding sources listed in your Funding Information later in the submission form are also declared in your Financial Disclosure statement.<br><br>View published research articles from *PLOS ONE* for specific examples.<br><br>**NO authors have competing interests**<br><br>Enter: *The authors have declared that no competing interests exist.*<br><br>**Authors with competing interests**<br><br>Enter competing interest details beginning with this statement:<br><br>*I have read the journal's policy and the authors of this manuscript have the following competing interests: [insert competing interests here]*<br><br>* typeset	
**Ethics Statement**<br><br>Enter an ethics statement for this submission. This statement is required if the study involved:<br><br>• Human participants<br>• Human specimens or tissue<br>• Vertebrate animals or cephalopods<br>• Vertebrate embryos or tissues<br>• Field research<br><br>Write "N/A" if the submission does not require an ethics statement.<br><br>General guidance is provided below. Consult the submission guidelines for detailed instructions. **Make sure that all information entered here is included in the Methods section of the manuscript.**	Fifty SD rats (6 weeks of age to 8 weeks of age) were purchased from Lanzhou University. The animal certificate number was 62000800000176 and animal license number was SCXK (Gan) 2013-0002. All animal experiments reported in this study were approved by the Ethics Committee of Gansu University of Chinese Medicine. The environment temperature was 222℃, the relative humidity was 5510%, and the environment alternates light and dark were 12 hours.

**Format for specific study types**<br><br>**Human Subject Research (involving human participants and/or tissue)**<br>• Give the name of the institutional review board or ethics committee that approved the study<br>• Include the approval number and/or a statement indicating approval of this research<br>• Indicate the form of consent obtained (written/oral) or the reason that consent was not obtained (e.g. the data were analyzed anonymously)<br><br>**Animal Research (involving vertebrate animals, embryos or tissues)**<br>• Provide the name of the Institutional Animal Care and Use Committee (IACUC) or other relevant ethics board that reviewed the study protocol, and indicate whether they approved this research or granted a formal waiver of ethical approval<br>• Include an approval number if one was obtained<br>• If the study involved *non-human primates*, add *additional details* about animal welfare and steps taken to ameliorate suffering<br>• If anesthesia, euthanasia, or any kind of animal sacrifice is part of the study, include briefly which substances and/or methods were applied<br><br>**Field Research**<br><br>Include the following details if this study involves the collection of plant, animal, or other materials from a natural setting:<br>• Field permit number<br>• Name of the institution or relevant body that granted permission	
**Data Availability**<br><br>Authors are required to make all data underlying the findings described fully available, without restriction, and from the time of publication. PLOS allows rare exceptions to address legal and ethical concerns. See the PLOS Data Policy and FAQ for detailed information.	Yes - all data are fully available without restriction

A Data Availability Statement describing where the data can be found is required at submission. Your answers to this question constitute the Data Availability Statement and **will be published in the article**, if accepted.<br><br>**Important:** Stating 'data available on request from the author' is not sufficient. If your data are only available upon request, select 'No' for the first question and explain your exceptional situation in the text box.<br><br>Do the authors confirm that all data underlying the findings described in their manuscript are fully available without restriction?	
**Describe where the data may be found in full sentences. If you are copying our sample text, replace any instances of XXX with the appropriate details.**<br><br>• If the data are **held or will be held in a public repository**, include URLs, accession numbers or DOIs. If this information will only be available after acceptance, indicate this by ticking the box below. For example: *All XXX files are available from the XXX database (accession number(s) XXX, XXX.).*<br>• If the data are all contained **within the manuscript and/or Supporting Information files**, enter the following: *All relevant data are within the manuscript and its Supporting Information files.*<br>• If neither of these applies but you are able to provide **details of access elsewhere**, with or without limitations, please do so. For example:<br><br>*Data cannot be shared publicly because of [XXX]. Data are available from the XXX Institutional Data Access / Ethics Committee (contact via XXX) for researchers who meet the criteria for access to confidential data.*<br><br>*The data underlying the results presented in the study are available from (include the name of the third party*	All relevant data are within the manuscript and its Supporting Information files.

and contact information or URL).<br>• This text is appropriate if the data are owned by a third party and authors do not have permission to share the data.<br><br>* typeset	
Additional data availability information:	

Manuscript Click here to access/download;Manuscript;Manuscript.docx

A potential marker of radiation based on 16S rDNA in the rat model: intestinal flora

Zhang Liying[1], Li Yangyang[1], Xu Xiaomin[1,2], Zhiming Miao[1], Zhou Ting[1], Zhang Yiming[1], Liu Yongqi [1,3*]

1. Provincial-Level Key Laboratory for Molecular Medicine of Major Diseases and The Prevention and Treatment with Traditional Chinese Medicine Research in Gansu Colleges and Universities, Gansu University of Chinese Medicine, Lanzhou 730000, Gansu, China;

2. Medical College of Hexi University, Zhangye 734000, Gansu, China;

3. Key Laboratory of Dunhuang Medicine and Transformation at Provincial and Ministerial Level, Gansu University of Chinese Medicine, Lanzhou 730000, Gansu, China

Abstract

The gastrointestinal microbiota plays an important role in the function of the host intestine. However, little is currently known about the effects of irradiation on the microorganisms colonizing the mucosal surfaces of the gastrointestinal tract. The aim of this study was to investigate the effects of X-ray irradiation on the compositions of the large intestinal microbiotas of the rat. The gut microbiotas in control mice and mice receiving irradiation with different dose treatment were characterized by high-throughput sequencing of the bacterial 16S rDNA gene and their metabolites were detected by gas chromatography-mass spectrometry. Unexpectedly, the diversity was increased mildly at 2Gy irradiation, and dose dependent decreased at 4Gy, 6Gy, 8Gy irradiation. The phyla with large changes in phylum level are Firmicutes, Bacteroides

and Proteobacteria; the abundance ratio of Firmicutes/Bacteroides is inverted; and when 8Gy is irradiated, the phylum abundance level was significantly increased. At the genus level, the abundance levels of *Phascolarctobacterium*, *Ruminococcaceae* and *Lachnospiraceae* increased at 2Gy irradiation, and significantly decreased at 4Gy, 6Gy, and 8Gy irradiation; the abundance level of *Prevotellaceae* diminished at 2Gy irradiation, and enhanced at 4Gy, 6Gy, 8Gy irradiation; The abundance level of *Violet bacteria* (*Christenellaceae*) and *Lactobacillus* attenuated in a dose-dependent manner; *Lachnoclostridium* enhanced in a dose-dependent manner; *Bacteroides* was in 4Gy, 6Gy, 8Gy The abundance level increased significantly during irradiation; the abundance level of *Shigella* (*Escherichia-Shigella*) only increased significantly during 8Gy irradiation. Lefse predicts that the biomarker at 0Gy group is *Veillonellaceae*, the biomarker at 2Gy group is *Firmicutes*, the biomarkers at 4Gy group are *Dehalobacterium* and *Dehalobacteriaceae*, the biomarkers at 6Gy group are *Odoribacter*, and the biomarkers at 8Gy group are *Anaerotruncus* , *Holdemania, Proteus, Bilophila, Desufovibrionales and Deltaproteobacteria*. Overall, the data presented here reveal that X-ray irradiation can cause imbalance of the intestinal flora in rats; different doses of irradiation can cause different types of bacteria change . Representative bacteria can be selected as biomarkers for radiation damage and repair.

Keywords: X-ray; radiation; 16S rDNA; intestinal flora dysbiosis

Introduction

Many studies have highlighted the presence of as many as 100 trillion bacterial

species in the human gut, which represent several times as many cell populations as somatic and germ cells in the human host[1][2][3]. These commensal bacteria of the gut are collectively called "gut microbiota," and increasing evidence suggests that the composition of gut microbiota is closely related to the health of the host, including the onset of disease[4][5]. Increasing evidence shows that the human gut microbiome plays a role in keeping the organism in a steady state, and intestinal dysbacteriosis is often accompanied by obesity[6], cardiovascular disease, inflammation disease[7], immune function disorder[8], metabolic disease[9][10][11] and even cancer[12]. Therefore, homeostasis of gut microbiota plays an important role in maintaining human health.

X-ray is one of the most used radiation therapies in clinic. After irradiation, it can cause DNA double strand or single strand breaks and other biological effects. It has been shown that the gut microbiota regulates the efficacy of radiotherapy and chemotherapy through a "TIMER" mechanism, which suggests a reduction in translocation, immune regulation, metabolism, enzymatic degradation, and diversity. Gut microbiota[13] and most tissue cells[14][15] in the body are sensitive to X-ray, especially those with active proliferation. Casero found that the mouse gut microbiome was response to ^{16}O radiation, dose-dependent changes in microflora diversity and composition of specific groups[16]. Zhang also shows that gut microbiome is very sensitive to IR and can be used as radiation biomarkers[17]. Recent reports suggest that metabolomics has shown increased concentrations of microbially derived propionic acid and tryptophan metabolites in feces of elite survivors, and use of these metabolites can lead to long-term radiation protection, relieve hematopoietic and gastrointestinal

syndromes, and reduce pro-inflammatory responses[18]. However, there are few reports about the effects of different doses of X-ray radiation on intestinal flora.

In this report, different doses of X-ray irradiation were used to detect the levels of intestinal flora and metabolites in feces of rats to observe the effects of different doses of X-ray irradiation on intestinal flora in SD rats.

Materials and Methods

Radiation

X-RAD-225 X-ray source (Precision, USA) was used to generate X-ray, the dose rate of which was about 200cGy/min (225.0 kV, 13.3 mA). The absorbed dose was 0Gy, 2Gy, 4Gy, 6Gy, 8Gy in each group. All irradiations were carried out at room temperature.

Animals and groups

Fifty SD rats (6 weeks of age to 8 weeks of age) were purchased from Lanzhou University. The animal certificate number was 62000800000176 and animal license number was SCXK (Gan) 2013-0002. All animal experiments reported in this study were approved by the Ethics Committee of Gansu University of Chinese Medicine. The environment temperature was 22±2℃, the relative humidity was 55±10%, and the environment alternates light and dark were 12 hours.

Fifty SD rats were randomly assigned into 0Gy, 2Gy, 4Gy, 6Gy, 8Gy groups (n=10 in each group). Rats in each group were treated with 0Gy, 2Gy, 4Gy, 6Gy, 8Gy dosses of X-ray respectively each time and the rats were sacrificed three days later after

radiation.

Microbiota Profiling

After the rats were sacrificed, midcolon contents were collected into several sterile EP tubes with corresponding numbers. The EP tubes were storaged in an ultra-low refrigerator at -80°C and transported with dry ice. Genome-wide DNA was extracted from midcolon contents to amplify using specific primers with barcodes (16S V3 + V4). Paired end sequencing was performed on the Illumina MiSeq Platform and analyzed according to the previous study[19]. We used principal coordinate analysis (PCoA) to obtain principal coordinates from complex multidimensional data and visualize them. Diversity and inhomogeneity indicators including Shannon, Simpson, Chao1, and ACE were detected on this platform[20]. OTUs were further used by PICRUST for genome prediction of microbial communities[21]. Each group was subjected to three biological verifications.

Gas chromatography-mass spectrometry

Preparation and High-Performance Liquid Chromatography (HPLC)-Based Analysis of FL on the Intestinal contents. 5g of mouse colon content was weighed and put into a beaker which was ground evenly after being dried in the shade, and 50mL methanol and a certain amount of sodium hydroxide was added and mixed until pH>=12. Then added 400mL water and 50mL dichloromethane in turn, vibrated for 5min and standed for 10min to separated the water phase and dichloromethane phase. The above solution was separated by the glass chromatographic column with 1g anhydrous sodium sulfate and 5g Florisil. Then added 20mL dichloromethane to elute

the chromatographic column, elute twice, combine the eluent for three times, rotate and evaporate, concentrate to nearly dry, and then fix the volume to 1mL with n-hexane. HPLC analyses were performed on a Shimadzu liquid chromatography system (Shimadzu LC-2030, Kyoto, Japan) equipped with an evaporative light detector (Shimadzu 228-4511538, Kyoto, Japan). Chromatographic separation of the analytes was performed on a SinoChrom ODS-BP C18 analytical column (LC Column, 250 mm × 4.6 mm, 5 μm). The sample injection volume was 10 μL.

Statistical Analysis

The one-way analysis of variance (ANOVA) was used to analysis the statistical data in three or more groups. Levene's test was carried out to test the homogeneity of variances and followed with student's T test between the two groups (SPSS 20.0 software). The Mean±SEM was used to represent the data and denoted as follows: ns P>0.05; *P<0.05; **P<0.01; ***P<0.001.

Result

Imaging results of agarose gel electrophoresis

The extract DNA of each mid colon contents sample was collected by PCR agarose gel electrophoresis. 15000bp bands can be seen in each sample, indicating that every sample is qualified (Fig1A). Agglomerate clustering is a hierarchical clustering algorithm. It is an unsupervised machine learning technique that partitions the population into several clusters, making data points more similar in the same cluster and different in different clusters. The result showed that the length of the branches of the same dose group was close and clustered together (Fig1B).

Radiation can reduce the number of OTUs

Operational Tax Units (OTUs) is often used in the analysis of microorganisms without culture, which can reverse the diversity and abundance of different microorganisms. Generally, if the similarity between sequences, such as different 16S rDNA sequences, is higher than 97%, it can be defined as an OTU. Fig2A-2B showed the result of the OTUs. The OTUs results showed that the number of OTUs in each group decreased after irradiation (Fig2A). There are 254 similar OTUs in different groups (Fig2B). There are 5 at 0Gy group different from the other groups, and there are 5 at 2Gy group, 3 at 4Gy group, 8 at 4Gy group, 3 at 8Gy group different from the other groups respectively (Fig2B).

Radiation can reduce the diversity of the intestinal flora

Alpha diversity (α diversity) refers to the diversity in a specific region or ecosystem, and the commonly used measurement indicators are as follows Shannon, Simpson, ace, Chao, etc. The greater the above index value, the higher the community diversity. Fig3A-3D showed the result of the Alpha diversity. The results showed that with the increase of irradiation dose, the α diversity of other groups decreased in a dose-dependent manner ($P<0.05$), and the α diversity of 6Gy and 8Gy groups decreased most obviously ($P<0.05$).

Beta diversity (β diversity) refers to the differences between samples, and the commonly used measurement indicators are as follows principal co-ordinates analysis (PCoA), Non-metric Multidimensional Scaling (NMDS) and Principal component analysis (PCA). PCoA is a visualization method to study the similarity or difference of

data, which can be used to observe the differences between individuals or groups. NMDS is often used to compare the differences between sample groups, which can be based on evolutionary relationship or quantitative distance matrix. PCA is a visual method to study the similarity or difference of data and it can find the most important coordinates in the distance matrix. Each point in the graph represents a sample, and the points with the same color come from the same group. The closer the distance between two points, the smaller the difference in community composition between them. Fig3E-3G showed the result of the β diversity. The results show that the distance between samples in 0Gy, 2Gy and 4Gy groups is close and difficult to distinguish, while samples in 6Gy and 8Gy occupy their own space and are easy to distinguish.

Radiation can change overall microbial composition in each group.

The overall microbial composition in each group differed at the phylum and genus levels (Fig4A-4N). The largest phylum represented in each dataset was *Firmicutes*, *Bacteroidetes*, and *Proteobacteria* (Fig4A, 4C-4E). The abundance level of *Firmicutes* enhanced when irradiated by 2Gy, but did not change obviously when irradiated by 4Gy and 6Gy (P>0.05), and attenuated when irradiated by 8Gy (P<0.05). The abundance level of *Bacteroidetes* decreased when irradiated by 2Gy, increased when irradiated by 4Gy and 6Gy, and attenuated when irradiated by 8Gy (P<0.05). The abundance ratio of *Firmicutes/Bacteroides* is inverted. The abundance level of *Proteobacteria* did not change obviously when irradiated by 2Gy, 4Gy and 6Gy (P>0.05), but increased significantly when irradiated by 8Gy (P<0.05).

At the genus level, the largest genus represented in each dataset was

Phascolarctobacterium, Ruminococcaceae, Christenellaceae, Lactobacillus, Prevotellaceae, Lachnospiraceae, Bacteroides, Lachnoclostridium and *Escherichia-Shigella* (Fig4B, 4F-4N). The abundance level of *Phascolarctobacterium Ruminococcaceae* and *Lachnospiraceae* increased at 2Gy, and decreased at 4Gy, 6Gy and 8Gy($P<0.05$). The abundance level of *Christenellaceae* and *Lactobacillus* decreased in a dose-dependent manner, while the abundance level of *Lachnoclostridium* increased in a dose-dependent manner. The abundance level of *Prevotellaceae* decreased at 2Gy, and increased at 4Gy, 6Gy and 8Gy ($P<0.05$). The abundance level of Bacteroides didn't change obviously at 2Gy ($P>0.05$), but increased significantly at 4Gy, 6Gy and 8Gy($P<0.05$). The abundance level of *Escherichia-Shigella* didn't change obviously at 2Gy, 4Gy and 6Gy ($P>0.05$), but increased significantly at 8Gy ($P<0.05$).

Biomarkers of intestinal flora at different irradiation doses

Lefse is based on linear discriminant analysis, which combines linear discriminant analysis with nonparametric Kruskal-Wallis and Wilcoxon rank sum test to screen Biomarker between groups (Fig5A-5B). It can be seen from the figure that the biomarker of 0Gy group is *Veillonellaceae*, the biomarker of 2Gy group is *Firmicutes*, the biomarker of 4Gy group is *Dehalobacterium* and *Dehalobacteriaceae*, and the biomarker of 6Gy group is *Odoribacter*. The biomarkers of 8Gy group are *Anaerotruncus, Holdemania, Proteus, Bilophila, Desufovibrionales* and *Deltaproteobacteria*.

Radiation can decrease the expression of the SCFAs in colon contents.

Short chain fatty acids include Acetic acid, propionic acid, and butyric acid mainly,

which were detected by gas chromatography-mass spectrometry. The abundance level of Acetic acid decreased in a dose-dependent manner, while the abundance level of propionic acid, and butyric acid decreased sharply when at 2Gy, 4Gy, 6Gy and 8Gy (Fig6A-6C). So radiation markedly reduced Acetic acid, propionic acid, and butyric acid.

Discussion

Radiotherapy is a common method for the treatment of malignant tumors, and X-ray is one of the most frequently used measure. However, it inevitably damages the normal tissues and intestinal homeostasis[22]. Intestinal symbiotic bacteria are a kind of bacteria that can resist the colonization of foreign microorganisms. They act together on the body to play a total defense function, which is called biological barrier. When the intestinal microecological homeostasis is out of balance, the colonization resistance of the intestinal tract to external pathogens decreases, which eventually leads to the invasion and pathogenicity of external bacteria, and even the potentially pathogenic bacteria in the intestinal tract begin to play their pathogenic role. The intestinal tract has the largest "reservoir" in the body. It can produce a large amount of endotoxin and enhance the damage induced by endotoxemia, significantly increasing the mortality of acute and critical diseases[23]. Previous studies have shown that radiation is one of the factors causing intestinal microecology imbalance[24]. In this experiment, we selected different doses of X-ray to radiate SD rats to evaluate the effect of different doses of X-ray on intestinal flora imbalance.

In this study, 15000bp bands can be seen in each sample (Fig1A) and the length of the branches of the same dose group was close and clustered together (Fig1B), indicating that every sample is qualified. The OTUs results showed that the number of OTUs decreased after irradiation at 6Gy and 8Gy (Fig2). Shannon, simpson, ace and chao1 indexes were examined for the alpha-diversity of the microbiomes. The above indexes were increased at 2Gy, decreased at 4Gy, 6Gy and 8Gy compared to controls (Fig3A-D). To assess overall differences in beta diversity, we applied NMDS, PCoA maps, and Pca spatial maps to weighted and unweighted UniFrac distance metric matrices generated for the sample set. Control and irradiated rats showed significant microbial community structure aggregation (Fig3E-G). This result further indicates that the type and abundance of intestinal microorganisms in rats can be changed from 6Gy total body irradiation dose, which is widely involved in various physiological activities of the body and is called "metagenomic" intestinal flora[17], and appears disorder at 6Gy and 8Gy irradiation dose. The intestinal flora response of low-dose and high-dose irradiation was not exactly the same. However, there is evidence showed that a higher richness and diversity in *B. dorsalis* flies[25] and a lower diversity in rats[16] and patients[26]. This is not exactly the same as our study, the reason maybe that different species have different responses to radiation.

As several previous studies on the mammalian gut have included human and ratio[27], *Bacteria phyla* and *Firmicutes w*ere identified as two major phyla in the small and large intestines of mice. Although there were differences in the abundance of multiple phyla between control and different dose irradiated groups, the abundance

ratio of *Firmicutes/Bacteroides* was opposite to that of control. Notably, *Proteobacteria* was identified in the irradiated large intestines at 8Gy, control samples and other dose samples, however, did not, suggesting that proliferation of microbial cells belonging to this phylum may increase after irradiation.The study have shown that *Proteobacteria* is the key microorganism to induce enteritis, which may be one of the markers of disease[28]. That means high doses radiation can induce the growth of pernicious bacteria.

At the genus level, 16sr RNA analysis showed that the abundance levels of *Phascolarctobacterium, Ruminococceae* and *Lachnospiraceae* decreased significantly at 4Gy, 6Gy and 8Gy. *Phascolarctobacterium* produce short chain fatty acids, which play a variety of important roles in maintaining human health, such as enhancing gastrointestinal function and reducing the level of inflammation[29]. The main metabolites of *Ruminococcaceae* are acetic acid and formic acid[30]. The bacteria in *Ruminococcaceae* mainly rely on absorption of monosaccharide and degradation of mucin to obtain energy. *Lachnospiraceae* may be a potential beneficial bacterium, which is involved in the metabolism of many carbohydrates[31]. The fermentation product acetic acid is the main source of energy for the host. The main metabolites of above three are Acetic acid propionic acid and Valeric acid, giving a hint that the decrease of short chain fatty acids may be related to the decrease of bacteria producing short chain fatty acids. *Lactobacillus* and *Christenellaceae* were considered as beneficial agents who are benevolent workers that help digest food[32], however the abundance levels of them radiation decreased in a dose-dependent manner compared

with control group. Studies have shown that *Lactobacillus* is beneficial to the health of the host, which can ferment sugars to produce lactic acid, maintain human health and regulate immune function[33]. *Christenellaceae* is beneficial to human health, and its abundance is higher in thinner people[34]. The abundance of *Prevotellaceae* increased with the irradiation of 4Gy, 6 Gy and 8Gy. Studies have shown that *Prevotellaceae* is one of the common bacteria causing endogenous infection, and the pathogenic substance may be collagenase, which is related to the decomposition of connective tissue. *Lachnoclostridium* is tend to exist in obese patients, it ascends in a dose-dependent manner after radiation[35]. Bacteroides are symbiotic bacteria in human intestines, which provide essential nutrients for the body[36]. However, when they enter other parts of the body other than the gastrointestinal tract, they will lead to the aggravation of abscess and other infections, and the abundance level will increase significantly at 4Gy, 6Gy and 8Gy irradiation. *Escherichia-Shigella* has been proved is the pathogen of dysentery and it is also one of the pathogenic bacteria that enter the blood after the destruction of intestinal barrier[37]. In this report, the abundance of Escherichia-Shigella was only dramatically elevated by radiation at 8Gy group. These three kinds of pathogenic bacteria showed a significant increase in different groups, unexpectedly the degree of their increase was different.

We have also identified some potential markers that may play a therapeutic role in different treatments. It was an interesting finding that the biomarkers of 2Gy group were *Firmicutes*, 4Gy group were *Dehalobacterium* and *Dehalobacteriaceae*, 6 Gy group were *Odoribacter*, 8Gy group were *Anaerotruncus, Holdemania, Proteus, Bilophila,*

Desufovibrionales and *Deltaproteobacteria.* This suggests that moxibustion and electroacupuncture may have some similar therapeutic targets. Although we have found some potential biomarkers, how to regulate these markers by moxibustion and electroacupuncture still requires further research. These findings indicate that the microbiota emerges as a critical driver of radiation-response, but still need to pose several questions. (1) There are only three samples in each group, so the sample size is too small. (2) how do specific bacterial species modulate inflammatory responses? (3) Whether these findings could translate into a therapeutic approach for the treatment of radiation-induced diseases in humans deserves further attention.

Funding

This study was funded by Natural Science Foundation of Gansu Province (NO. 20JR10RA332, 20JR10RA318). National Natural Science Foundation of China (NO. 82260882, 82004094). China Postdoctoral Science Foundation Project (NO. 2021M693794). Lanzhou City Health Key Science and Technology Development Project (NO. 2021006).

Declarations

Not applicable

Reference

[1] Bäckhed F, Ding H, Wang T, et al. The gut microbiota as an environmental facto r that regulates fat storage [J]. Proc Natl Acad Sci U S A. 2004,101(44):15718-1 5723.

[2] Hooper LV, Macpherson AJ. Immune adaptations that maintain homeostasis with t

he intestinal microbiota [J]. Nat Rev Immunol. 2010,10(3):159-169.

[3] Bonder MJ, Kurilshikov A, Tigchelaar EF, et al. The effect of host genetics on the gut microbiome [J]. Nat Genet. 2016,48(11):1407-1412.

[4] Clements SJ, R Carding S. Diet, the intestinal microbiota, and immune health in aging [J]. Crit Rev Food Sci Nutr. 2018,4(4):651-661.

[5] Albenberg LG, Wu GD. Diet and the intestinal microbiome: associations, functions, and implications for health and disease [J]. Gastroenterology. 2014,146(6):1564-1572.

[6] Yin J, Li Y, Han H, et al. Melatonin reprogramming of gut microbiota improves lipid dysmetabolism in high-fat diet-fed mice [J]. J Pineal Res. 2018,65(4):e12524.

[7] Laurent S, Bruno RM. Gut microbiome composition, a third player in the inflammation-arterial stiffness relationship [J]. Eur Heart J. 2018,39(25):2398-2400.

[8] Lei YM, Nair L, Alegre ML. The interplay between the intestinal microbiota and the immune system [J]. Clin Res Hepatol Gastroenterol. 2015,39(1):9-19.

[9] Zhernakova A, Kurilshikov A, Bonder MJ, et al. Population-based metagenomics analysis reveals markers for gut microbiome composition and diversity. Science. 2016,352(6285):565-569.

[10] Zhang Y, Lu S, Yang Y, et al. The diversity of gut microbiota in type 2 diabetes with or without cognitive impairment [J]. Aging Clin Exp Res. 2021,33(3):589-601.

[11] Karlsson FH, Tremaroli V, Nookaew I, et al. Gut metagenome in European women with normal, impaired and diabetic glucose control [J]. Nature. 2013,498(7452):99-103.

[12] Libertucci J, Young VB. The role of the microbiota in infectious diseases. Nat Microbiol[J]. 2019,4(1):35-45.

[13] Wang Z, Wang Q, Wang X, et al. Gut microbial dysbiosis is associated with development and progression of radiation enteritis during pelvic radiotherapy [J]. J Cell Mol Med. 2019,23(5):3747-3756.

[14] Tajima G, Delisle AJ, Hoang K, et al. Immune system phenotyping of radiation a

nd radiation combined injury in outbred mice[J]. Radiat Res. 2013,179(1):101- 112.

[15] Lopetuso L R, Petito V, Scaldaferri F, et al. Gut microbiota modulationand mucosal immunity: Focus on rifaximin[J]. Mini Rev MedChem.2015,16(3):179-185.

[16] David C, Kirandeep G, Vijayalakshmi S, et al. Space-type radiation induces multimodal responses in themouse gut microbiome and metabolome[J]. Microbiome. 2017;5:105.

[17] Zhang A, Steen TY. Gut Microbiomics-A Solution to Unloose the Gordian Knot of Biological Effects ofIonizing Radiation[J]. J Hered. 2017:109:212-221.

[18] Guo H, Chou WC, Lai Y, et al. Multi-omics analyses of radiation survivors identify radioprotective microbes and metabolites [J]. Science. 2020,370(6516):e9097.

[19] Zhang X, Zhao Y, Zhang M, et al. Structural changes of gut microbiota during berberine-mediated prevention of obesity and insulinresistance in high-fat diet-fed rats [J]. PLoS One. 2012;7:e42529.

[20] Caporaso JG, Kuczynski J, Stombaugh, et al. QIIME allowsanalysis of high-throughput community sequencingdata [J]. Nat Methods. 2010;7:335-336.

[21] Lozupone C, Knight R. UniFrac: a new phylogeneticmethod for comparing microbial communities [J]. Appl Environ Microbiol. 2005;71:8228-8235.

[22] Viggiano D, Ianiro G, Vanella G, et al. Gut barrier in health and disease: focus on childhood [J]. Eur Rev Med Pharmacol Sci. 2015,19(6):1077-1085.

[23] Nagpal I, Abraham SK. Protective effects of tea polyphenols and β-carotene against γ-radiation induced mutation and oxidative stress in Drosophila melanogaster [J]. Genes Environ. 2017,39(1):24.

[24] Wilmore JR, Gaudette BT, Gomez Atria D, et al. Commensal Microbes Induce Serum IgA Responses that Protect against Polymicrobial Sepsis [J]. Cell Host Microbe. 2018,23(3):302- 311.

[25] Cai ZH, Yao ZC, Li YS, et al. Intestinal probiotics restore the ecological fitness decline of Bactrocera dorsalis by irradiation [J]. Evol Appl. 2018,11(10):1946-1963.

[26] Wang Z, Wang Q, Wang X, et al. Gut microbial dysbiosis is associated with dev

elopment and progression of radiation enteritis during pelvic radiotherapy [J]. J Cell Mol Med. 2019,23(5):3747-3756.

[27] Ivanov II, Honda K. Intestinal commensal microbes as immune modulators [J]. Cell Host Microbe. 2012,12(4):496-508.

[28] Shin NR, Whon TW, Bae JW. Proteobacteria: microbial signature of dysbiosis in gut microbiota [J]. Trends Biotechnol. 2015,33(9):496-503.

[29] Zhang J, Guo Z, Xue Z, et al. A phylo-functional core of gut microbiota in healthy young Chinese cohorts across lifestyles, geography and ethnicities [J]. ISME J. 2015,9(9):1979-90.

[30] Hahnke S, Abendroth C, Langer T, et al. Complete Genome Sequence of a New Ruminococcaceae Bacterium Isolated from Anaerobic Biomass Hydrolysis [J]. Genome Announc. 2018,6(14):e00030-18

[31] Sorbara MT, Littmann ER, Fontana E, et al. Functional and Genomic Variation between Human-Derived Isolates of Lachnospiraceae Reveals Inter- and Intra-Species Diversity [J]. Cell Host Microbe. 2020,28(1):134-146.

[32] Talib N, Mohamad NE, Yeap SK, et al. Isolation and Characterization of Lactobacillus spp. from Kefir Samples in Malaysia. Molecules. 2019,24(14):2606.

[33] Vojinovic D, Radjabzadeh D, Kurilshikov A, et al. Relationship between gut microbiota and circulating metabolites in population-based cohorts [J]. Nat Commun. 2019,10(1):5813.

[34] Cuevas-Sierra A, Riezu-Boj JI, Guruceaga E, et al. Sex-Specific Associations between Gut Prevotellaceae and Host Genetics on Adiposity [J]. Microorganisms. 2020,8(6):938.

[35] Ocvirk S, Wilson AS, Posma JM, et al. A prospective cohort analysis of gut microbial co-metabolism in Alaska Native and rural African people at high and low risk of colorectal cancer [J]. Am J Clin Nutr. 2020,111(2):406-419.

[36] Wexler HM. Bacteroides: the good, the bad, and the nitty-gritty [J]. Clin Microbiol Rev. 2007,20(4):593-621.

[37] Bin P, Tang Z, Liu S, et al. Intestinal microbiota mediates Enterotoxigenic Escherichia coli-induced diarrhea in piglets [J]. BMC Vet Res. 2018,14(1):385.

Figure legend

Figure 1: homogeneity detection of samples. PCR agarose gel electrophoresis was used to detected of V3 and V4 protein content. We can see the 15000bp bands in each sample (Fig1A). The cluster analysis was provided by R software and the results of all samples are shown in the Fig1B.

Figure 2: Radiation can reduce the number of OTUs. Fig2A shows the comparative Wayne diagram of OTUs composition. Fig2B shows the number of OTUs in each group after irradiation. Values are presented as mean±SEM, n=3. Differences were assessed by ANOVA for multiple comparisons and denoted as follows: ns P>0.05; *P<0.05; **P<0.01; ***P<0.001.

Figure 3: Radiation can reduce the diversity of the intestinal flora. Fig3A showed the result of the Shannon index in α-diversity analysis. Fig3B showed the result of the Simpson index in α-diversity analysis. Fig3C showed the result of the ace index in α-diversity analysis. Fig3D showed the result of the chao index in α-diversity analysis. Fig3E showed the result of the NMDS index in β-diversity analysis. Fig3F showed the result of the chao index in β-diversity analysis. Fig3G showed the result of the chao index in β-diversity analysis. Values are presented as mean±SEM, n=3. Differences were assessed by ANOVA for multiple comparisons and denoted as follows: ns P>0.05; *P<0.05; **P<0.01; ***P<0.001.

Figure 4: Radiation can change overall microbial composition in each group by X ray. Microbiota compositions at the phylum level (Fig4A) and at the genus level

(Fig4B) have been shown in this figure. The figures show bacteria with obvious changes at the phylum level (Fig4C-4E). The figures show bacteria with obvious changes at the genus level (Fig4F-4N). Values are presented as mean±SEM. Differences were assessed by ANOVA for multiple comparisons and denoted as follows: ns $P>0.05$; *$P<0.05$; **$P<0.01$; ***$P<0.001$.

Figure 5 : The analysis of LDA difference contribution interpretation of the map. Different colors in the map represent significantly different species between different samples or groups. It is obtained by analysis with LefSe software, in which the significant difference of the logarithmic LDA score is set to 2.

Figure 6 : Radiation can decrease the expression of the SCFAs in colon contents. Acetic acid (Fig6A), propionic acid (Fig6B), and butyric acid (Fig6C) were detected by gas chromatography-mass spectrometry. Values are presented as mean±SEM. Differences were assessed by ANOVA for multiple comparisons and denoted as follows: ns $P>0.05$; *$P<0.05$; **$P<0.01$; ***$P<0.001$.

Figure Click here to access/download;Figure;Fig 9.1.docx

Fig1

A

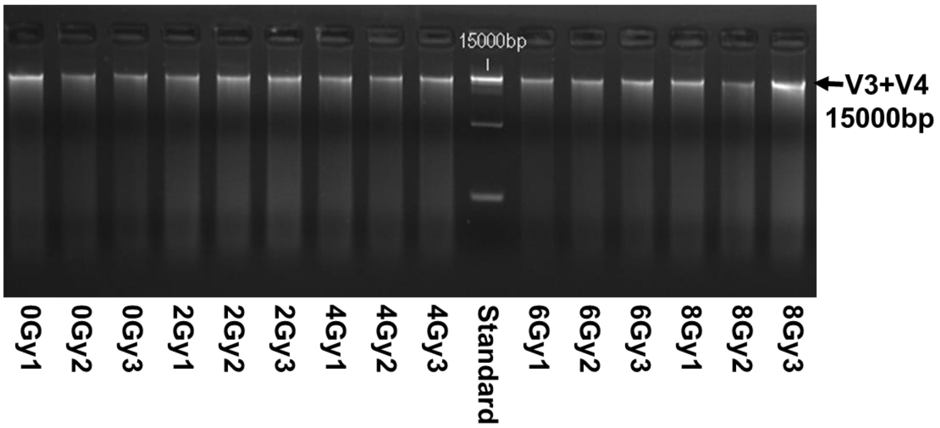

B

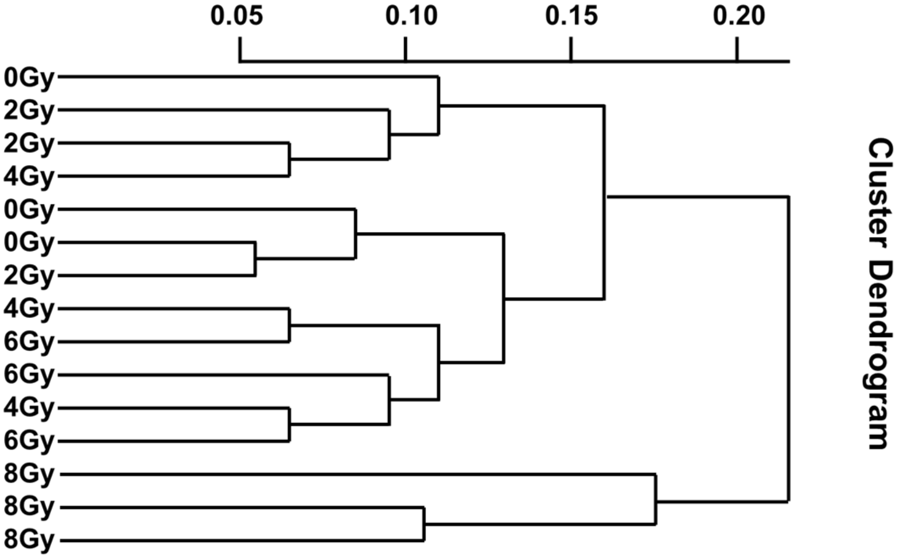

Fig2

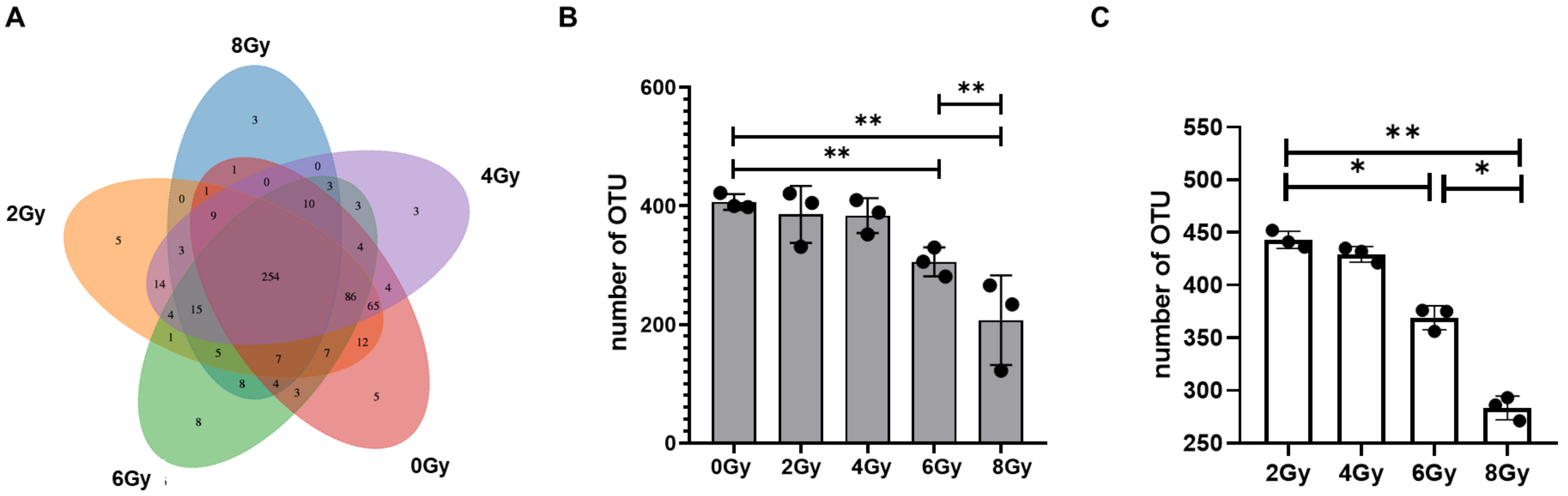

A
8Gy
4Gy
2Gy
6Gy
0Gy
254
B
number of OTU
600
400
200
0
0Gy
2Gy
4Gy
6Gy
8Gy
**
**
**
C
number of OTU
550
500
450
400
350
300
250
2Gy
4Gy
6Gy
8Gy
**
*
*

Fig3

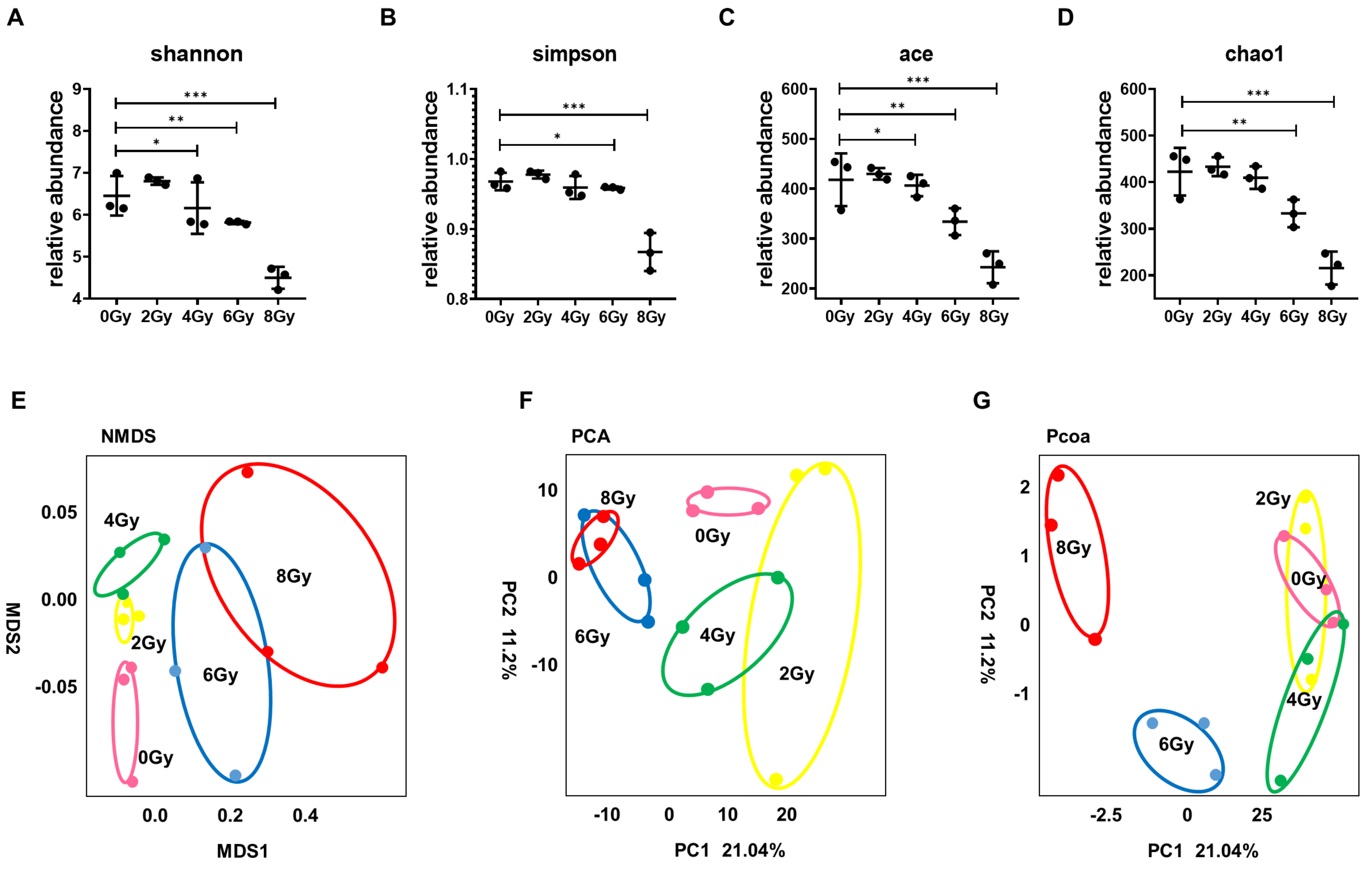
A
shannon
relative abundance
9
8
7
6
5
4
0Gy 2Gy 4Gy 6Gy 8Gy
***
**
*
B
simpson
relative abundance
1.1
1.0
0.9
0.8
0Gy 2Gy 4Gy 6Gy 8Gy
***
*
C
ace
relative abundance
600
500
400
300
200
0Gy 2Gy 4Gy 6Gy 8Gy
***
**
*
D
chao1
relative abundance
600
500
400
300
200
0Gy 2Gy 4Gy 6Gy 8Gy
***
**
E
NMDS
MDS2
0.05
0.00
-0.05
4Gy
2Gy
0Gy
6Gy
8Gy
0.0
0.2
0.4
MDS1
F
PCA
PC2 11.2%
10
0
-10
8Gy
0Gy
6Gy
4Gy
2Gy
-10
0
10
20
PC1 21.04%
G
Pcoa
PC2 11.2%
2
1
0
-1
8Gy
2Gy
0Gy
4Gy
6Gy
-2.5
0
25
PC1 21.04%

Fig4

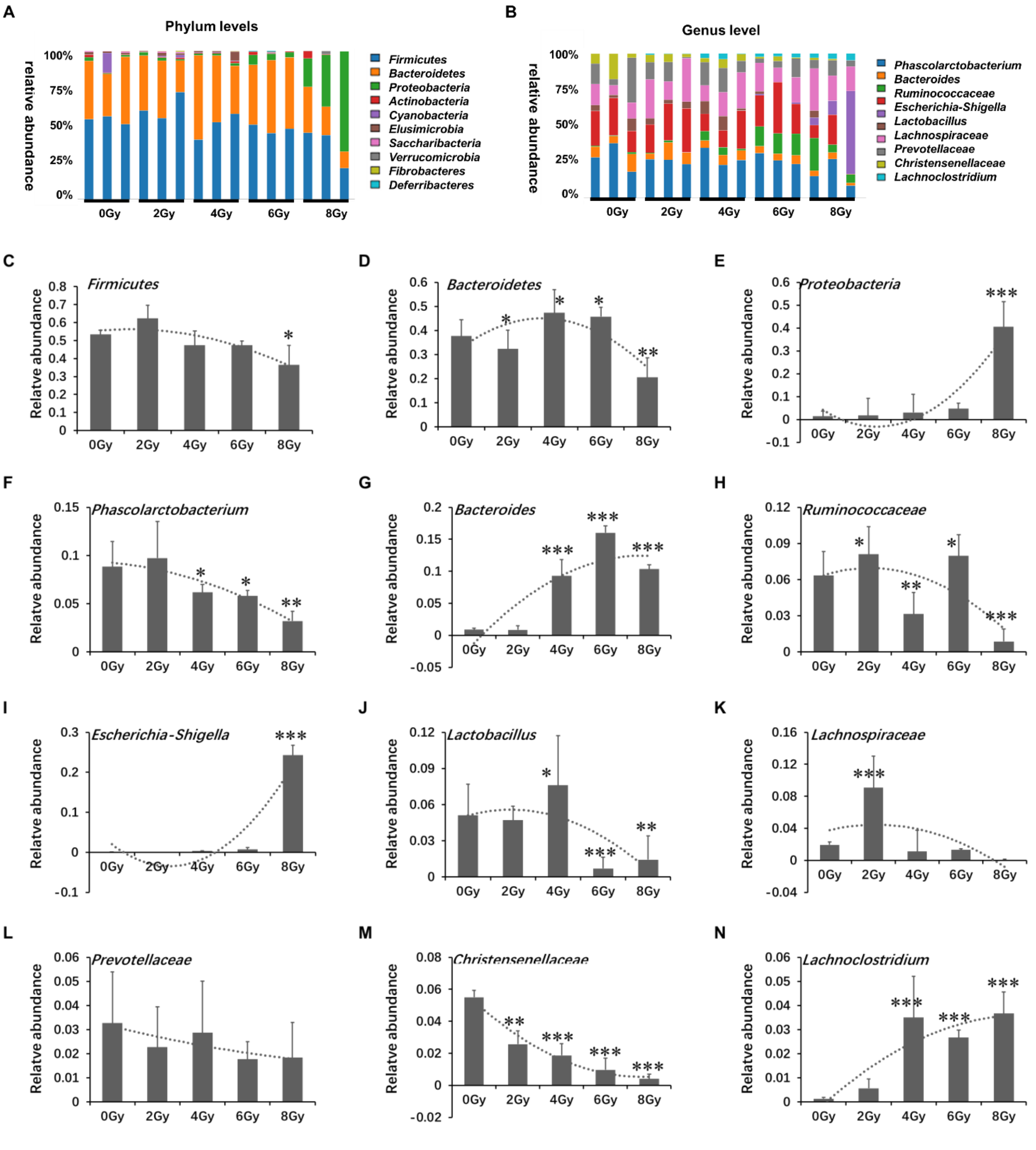
A
Phylum levels
relative abundance
100%
75%
50%
25%
0%
0Gy
2Gy
4Gy
6Gy
8Gy
Firmicutes
Bacteroidetes
Proteobacteria
Actinobacteria
Cyanobacteria
Elusimicrobia
Saccharibacteria
Verrucomicrobia
Fibrobacteres
Deferribacteres
B
Genus level
Phascolarctobacterium
Bacteroides
Ruminococcaceae
Escherichia-Shigella
Lactobacillus
Lachnospiraceae
Prevotellaceae
Christensenellaceae
Lachnoclostridium
C
Firmicutes
D
Bacteroidetes
E
Proteobacteria
F
Phascolarctobacterium
G
Bacteroides
H
Ruminococcaceae
I
Escherichia-Shigella
J
Lactobacillus
K
Lachnospiraceae
L
Prevotellaceae
M
Christensenellaceae
N
Lachnoclostridium
Relatve abundance

Fig5

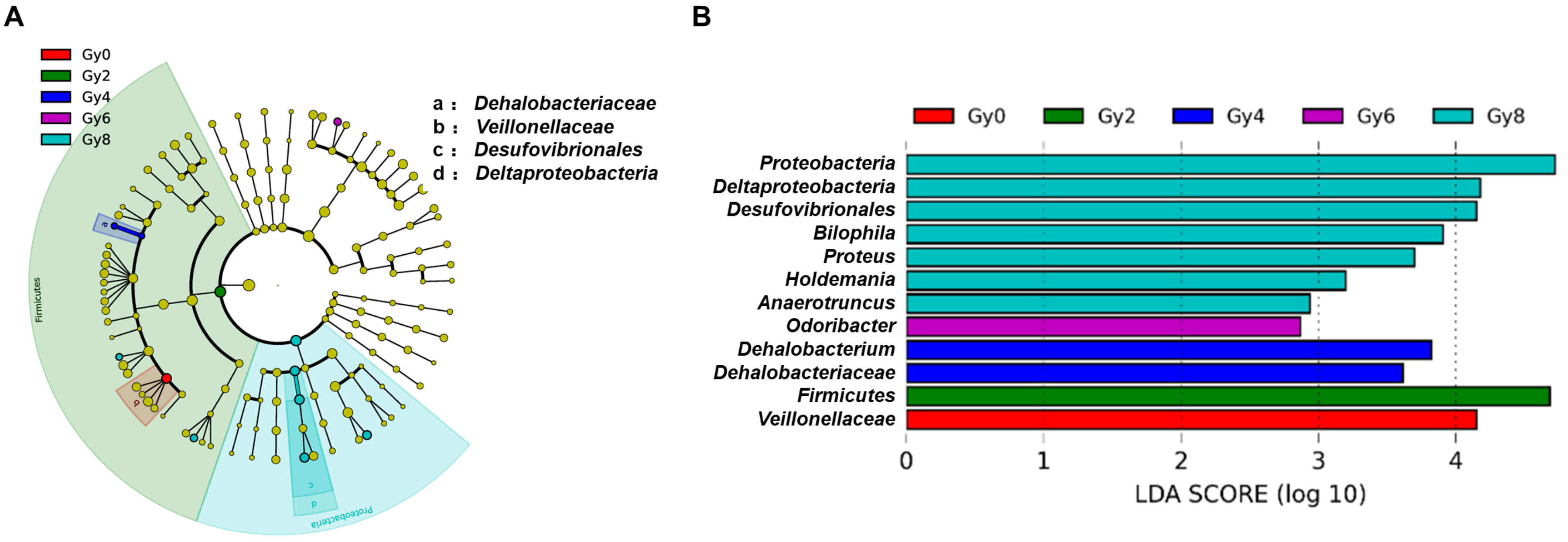
A
Gy0
Gy2
Gy4
Gy6
Gy8
Firmicutes
Proteobacteria
a : Dehalobacteriaceae
b : Veillonellaceae
c : Desufovibrionales
d : Deltaproteobacteria
B
Gy0
Gy2
Gy4
Gy6
Gy8
Proteobacteria
Deltaproteobacteria
Desufovibrionales
Bilophila
Proteus
Holdemania
Anaerotruncus
Odoribacter
Dehalobacterium
Dehalobacteriaceae
Firmicutes
Veillonellaceae
0
1
2
3
4
LDA SCORE (log 10)

Fig6

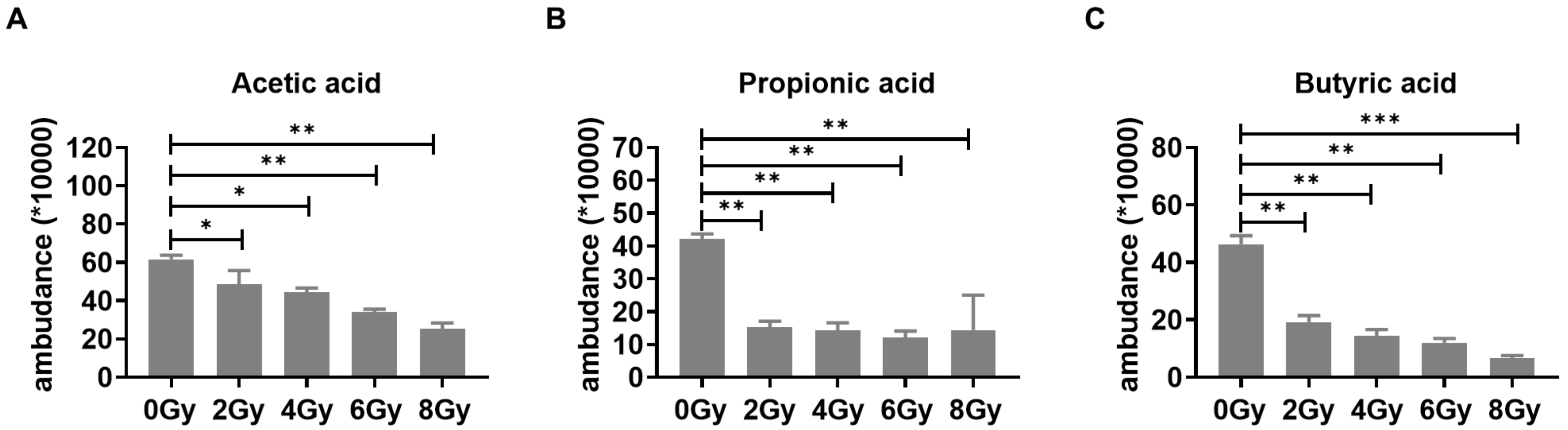
A
Acetic acid
ambudance (*10000)
0
20
40
60
80
100
120
0Gy
2Gy
4Gy
6Gy
8Gy
*
*
**
**
B
Propionic acid
ambudance (*10000)
0
10
20
30
40
50
60
70
0Gy
2Gy
4Gy
6Gy
8Gy
**
**
**
**
C
Butyric acid
ambudance (*10000)
0
20
40
60
80
0Gy
2Gy
4Gy
6Gy
8Gy
**
**
**
***
