# Supplementary material for: A potential marker of radiation based on 16S rDNA in the rat model: Intestinal flora
Source: PLoS One. 2023 Aug 1;18(8):e0286026. doi: 10.1371/journal.pone.0286026 (PMC10393172; doi:10.1371/journal.pone.0286026)
Supplement: Attachment — Submitted filename: Response to Reviewers.docx [file pone.0286026.s003.docx]

Dear editor,

Thank you for your kind considerations on our manuscript (PONE-D-22-28249). I am writing concerning the revision deadline of the obove mentioned manuscript.

The revision work for the manuscript is not complete yet. Because the revision information we received on March 27, we are unable to submit the manuscript on time.

Could you please extend the deadline to April 20 2023? So that we can revise the manuscript more appropriately.

Thanks very much for your help!

Regards,

Liying Zhang

Journal requirements:

When submitting your revision, we need you to address these additional requirements.

1. Please ensure that your manuscript meets PLOS ONE's style requirements, including those for file naming. The PLOS ONE style templates can be found at

https://journals.plos.org/plosone/s/file?id=wjVg/PLOSOne_formatting_sample_main_body.pdf and <https://journals.plos.org/plosone/s/file?id=ba62/PLOSOne_formatting_sample_title_authors_affiliations.pdf>.

Hello, editor, we have changed it according to the format.

2. To comply with PLOS ONE submissions requirements, in your Methods section, please provide additional information regarding the experiments involving animals and ensure you have included details on (1) methods of sacrifice, (2) methods of anesthesia and/or analgesia, and (3) efforts to alleviate suffering.

Mice were anesthetized with pentobarbital sodium 50 mg/kg by intraperitoneal injection, which provided adequate sedation and analgesia, and then cervically dislocated to ensure their euthanasia.

3. We noticed you have some minor occurrence of overlapping text with the following previous publication(s), which needs to be addressed:

- https://pubmed.ncbi.nlm.nih.gov/25600706/#:~:text=The%20gut%20microbiotas%20in%20control,intestines%20at%20the%20genus%20level.

- https://www.hindawi.com/journals/ecam/2020/9017854/

In your revision ensure you cite all your sources (including your own works), and quote or rephrase any duplicated text outside the methods section. Further consideration is dependent on these concerns being addressed.

Hello, Editor, this part has been modified in the text. We mainly have some overlap in the experimental methods, and our experiments as well as the papers are original. Thank you for your comments.

4. We note that the grant information you provided in the ‘Funding Information’ and ‘Financial Disclosure’ sections do not match.

When you resubmit, please ensure that you provide the correct grant numbers for the awards you received for your study in the ‘Funding Information’ section.

Hello Editor, this part has been modified upon submission. Thank you for your comments.

5. Thank you for stating the following in the Acknowledgments/ Funding Section of your manuscript:

“This study was funded by Natural Science Foundation of Gansu Province (NO. 20JR10RA332, 20JR10RA318). National Natural Science Foundation of China (NO. 82260882, 82004094). China Postdoctoral Science Foundation Project (NO. 2021M693794). Lanzhou City Health Key Science and Technology Development Project (NO. 2021006).”

We note that you have provided funding information that is not currently declared in your Funding Statement. However, funding information should not appear in the Acknowledgments section or other areas of your manuscript. We will only publish funding information present in the Funding Statement section of the online submission form.

Please remove any funding-related text from the manuscript and let us know how you would like to update your Funding Statement. Currently, your Funding Statement reads as follows:

“NO - Include this sentence at the end of your statement: The funders had no role in study design, data collection and analysis, decision to publish, or preparation of the manuscript.”

Please include your amended statements within your cover letter; we will change the online submission form on your behalf.

Hello Editor, this part has been modified upon submission. It was our error in submitting it. Thank you for your advice.

6. Thank you for stating the following in your Competing Interests section:

“NO authors have competing interests”

Please complete your Competing Interests on the online submission form to state any Competing Interests. If you have no competing interests, please state ""The authors have declared that no competing interests exist."", as detailed online in our guide for authors at http://journals.plos.org/plosone/s/submit-now

 This information should be included in your cover letter; we will change the online submission form on your behalf.

Hello Editor, this part has been modified upon submission. Thank you for your suggestions.

7. PLOS ONE now requires that authors provide the original uncropped and unadjusted images underlying all blot or gel results reported in a submission’s figures or Supporting Information files. This policy and the journal’s other requirements for blot/gel reporting and figure preparation are described in detail at https://journals.plos.org/plosone/s/figures#loc-blot-and-gel-reporting-requirements and https://journals.plos.org/plosone/s/figures#loc-preparing-figures-from-image-files. When you submit your revised manuscript, please ensure that your figures adhere fully to these guidelines and provide the original underlying images for all blot or gel data reported in your submission. See the following link for instructions on providing the original image data: https://journals.plos.org/plosone/s/figures#loc-original-images-for-blots-and-gels.

In your cover letter, please note whether your blot/gel image data are in Supporting Information or posted at a public data repository, provide the repository URL if relevant, and provide specific details as to which raw blot/gel images, if any, are not available. Email us at [plosone@plos.org](mailto:plosone@plos.org) if you have any questions.

Hello editor, our article does not involve blots as well as gel images. Thank you for your suggestions.

8. Please review your reference list to ensure that it is complete and correct. If you have cited papers that have been retracted, please include the rationale for doing so in the manuscript text, or remove these references and replace them with relevant current references. Any changes to the reference list should be mentioned in the rebuttal letter that accompanies your revised manuscript. If you need to cite a retracted article, indicate the article’s retracted status in the References list and also include a citation and full reference for the retraction notice.

Hello editor, our article does not cover this kind of reference. Thank you for your suggestions.

Reviewer #1: Reviewer comments are attached in the manuscript pdf file. overall, authors performed a rigorous technic and methodology. A a further explanation and discussion are required. Additional figure to describ the study design is needed.

Due to the need to upload the reply to the reviewer separately, we have sorted out and answered the opinions of the reviewer below.

1. In the method section, please add the statistical method information that had been used in the study in concisely.

Hello, reviewer, thank you for your comments. In the text, we introduce the statistical analysis. In order to prevent excessive abstract word count, the statistical analysis was not described in the abstract.

2. Please add one sentence for recommendation based on the findings of the study.

Hello, reviewer, thank you for your comments, which have been revised in the original text.

3. Grouping of SD rats should be shown in figure.

Hello reviewer, thank you for your opinion. The grouping here is consistent with the grouping in the following text.

4. What is EP stand for?

Hello reviewer, EP tubes means a microcentrifuge tube.

5. where this activity was performed?

Dear reviewer, thank you for your question. We operated in the professional animal materials room of Gansu University of Traditional Chinese Medicine.

6. where the conclusion section is?

Hello reviewer, thank you for your question. Since our paper contains many results, we integrated the conclusion with the discussion. Written in the discussion section.

Reviewer #2: Recent study in microbiome is just providing insight into the composition, pattern, and predict functions of the intestinal microbiome in SD rats, and certain abundant genera and species could serve as a biomarker for diagnosis and treatment. in this regard this MS is timely and valuble.

1. Requires edition> Example: Abstract part (line 5. Microbiotas. Punctuation: line. 28 change.). Ethics statement: line 5. 222℃. EP tubes. it is not usual, to begin with, a number (5g of a mouse). (Five grams of…). SD rats. In its first use: Sprague Dawley rates.

Hello, reviewer, thank you for your comments, which have been revised in the original text.

2. Introduction. After irradiation, it can cause DNA double-strand or single-strand breaks and other biological effects. It has been shown that the gut microbiota regulates the efficacy of radiotherapy and chemotherapy through a "TIMER" mechanism, which suggests a reduction in translocation, immune regulation, metabolism, enzymatic degradation, and diversity. Requires citation.

. Casero found … David C et al in 2017 found...

. Zhang also shows…. Zhang A and Steen TY in 2017 also showed...

Hello, reviewer, thank you for your comments, which have been revised in the original text.

3. They act together on the body to play a total defense function, which is called a biological barrier. .... begin to play their pathogenic role. Citation Required.

Hello, reviewer, thank you for your comments, which have been revised in the original text.

4. Result From 16S rDNA sequencing: How many counts were generated and what % of proportion clustered with similarity indicated unique OTUs?

Hello reviewer, our calculated number is 547.Such as different 16S rDNA sequences, is higher than 97%, it can be defined as an OTU.

5. Your mention of the “metagenomic" intestinal flora is not clear for readers and requires further clear writing. As several previous studies on the mammalian gut have included humans and ratio [27] clarify.

Hello, reviewer, thank you for your comments, which have been revised in the original text.

6. These could be the limitations of the study

(1) There are only three samples in each group, so the sample size is too small. (2) how do specific bacterial species modulate inflammatory responses?

Hello reviewer, thank you for your comments, (1) we wrote 3 for the 3 animals, but we had 9 mice in each group during the experiment.(2) The content of this part will be explored in the following experiment. Thank you for your advice, which is very helpful to us.

7. This could be your recommendation: (3) Whether these findings could translate into a therapeutic approach for the treatment of radiation-induced diseases in humans deserves further attention.

Hello, reviewer, thank you for your suggestions. We are also thinking about whether suitable natural drugs can be found to target the results of this paper. Your advice is very helpful to us, and we will think about it seriously and explore it in the following work.
